# Tracking the Cognitive, Social, and Neuroanatomical Profile in Early Neurodegeneration: Type III Cockayne Syndrome

**DOI:** 10.3389/fnagi.2013.00080

**Published:** 2013-11-26

**Authors:** Sandra Baez, Blas Couto, Eduar Herrera, Yamile Bocanegra, Natalia Trujillo-Orrego, Lucia Madrigal-Zapata, Juan Felipe Cardona, Facundo Manes, Agustin Ibanez, Andres Villegas

**Affiliations:** ^1^Laboratory of Experimental Psychology & Neuroscience (LPEN), Institute of Cognitive Neurology (INECO) & Institute of Neuroscience, Favaloro University, Buenos Aires, Argentina.; ^2^National Scientific and Technical Research Council (CONICET), Buenos Aires, Argentina; ^3^Pontifical Catholic University of Argentina, Buenos Aires, Argentina; ^4^UDP-INECO Foundation Core on Neuroscience (UIFCoN), Diego Portales University, Santiago, Chile; ^5^Universidad Autónoma del Caribe, Barranquilla, Colombia; ^6^Facultad de Psicología, Universidad de San Buenaventura, Medellín, Colombia; ^7^Neuroscience Research Program, University of Antioquia, Medellín, Colombia; ^8^Australian Research Council (ARC) Centre of Excellence in Cognition and its Disorders, Canberra, ACT, Australia

**Keywords:** Cockayne syndrome, *ERCC8*, social cognition, cognitive profile, executive functions, VBM, early-onset neurodegeneration

## Abstract

Cockayne syndrome (CS) is an autosomal recessive disease associated with premature aging, progressive multiorgan degeneration, and nervous system abnormalities including cerebral and cerebellar atrophy, brain calcifications, and white matter abnormalities. Although several clinical descriptions of CS patients have reported developmental delay and cognitive impairment with relative preservation of social skills, no previous studies have carried out a comprehensive neuropsychological and social cognition assessment. Furthermore, no previous research in individuals with CS has examined the relationship between brain atrophy and performance on neuropsychological and social cognition tests. This study describes the case of an atypical late-onset type III CS patient who exceeds the mean life expectancy of individuals with this pathology. The patient and a group of healthy controls underwent a comprehensive assessment that included multiple neuropsychological and social cognition (emotion recognition, theory of mind, and empathy) tasks. In addition, we compared the pattern of atrophy in the patient to controls and to its concordance with *ERCC8* gene expression in a healthy brain. The results showed memory, language, and executive deficits that contrast with the relative preservation of social cognition skills. The cognitive profile of the patient was consistent with his pattern of global cerebral and cerebellar loss of gray matter volume (frontal structures, bilateral cerebellum, basal ganglia, temporal lobe, and occipito-temporal/occipito-parietal regions), which in turn was anatomically consistent with the *ERCC8* gene expression level in a healthy donor’s brain. The study of exceptional cases, such as the one described here, is fundamental to elucidating the processes that affect the brain in premature aging diseases, and such studies provide an important source of information for understanding the problems associated with normal and pathological aging.

## Introduction

Cockayne syndrome (CS) is a rare autosomal recessive disease that is related to defective DNA transcription or repair and cellular hypersensitivity to ultraviolet light (UV) (Laugel, 2013). Its two hallmarks are profound postnatal growth failure of the soma and brain, which are associated with premature aging and progressive multiorgan degeneration (Rapin et al., 2000). This condition is also characterized by cachexia, dementia, vision and hearing loss, cutaneous photosensitivity without propensity to cancer, endocrinopathies, progressive spasticity, ataxia, peripheral neuropathy, weakness, osteopenia, and joint contractures (Hanawalt, 2000; Rapin et al., 2000, 2006). CS is a progressive disorder, and most symptoms typically appear in early childhood and worsen over time (Rapin et al., 2006). All CS patients show similar symptoms, but the time of onset and the rate of progression vary among the subtypes (Laugel, 2013).

Clinically, this syndrome has been classified into three severity groups: classical CS/type I, severe infantile form/type II, and a milder subtype/type III (Ghai et al., 2011; Natale, 2011). In the current study we present the case of a patient with type III CS. This subtype may only manifest the first symptoms after adolescence and it is characterized by cognitive impairment, progressive cerebellar symptoms, and hearing loss. Type III CS patients might have mild intellectual disability and learning difficulties in primary school. They are usually diagnosed in early adolescence, and the mean life expectancy is approximately 30 years (Ghai et al., 2011; Laugel, 2013). Rare cases of adult-onset CS have been identified (Hashimoto et al., 2008); these patients may be photosensitive and may also show one or more symptoms associated with CS (Natale, 2011). In these cases, early dementia is often reported, typically after 30 years of age (Laugel, 2013).

Cockayne syndrome can be caused by mutations in either of two genes, *ERCC8* or *ERCC6*, located on chromosomes 5 and 10, respectively (Hanawalt, 2000; Spivak, 2004). The patient described here carries one of the 30 different mutations identified in the *ERCC8* gene (Laugel, 2013). The clinical spectra of these patients’ phenotypes are largely overlapping, and neither the site nor the nature of the mutation correlates with the severity of the clinical features (Laugel et al., 2010; Laugel, 2013). However, mutations associated with this gene seem to be preferentially linked to types I and III (Laugel et al., 2010).

Neuroimaging studies have shown that cerebral and cerebellar atrophy, brain calcifications, and white matter abnormalities are cardinal features of CS (Koob et al., 2010; Laugel, 2013). White matter loss, ventricular enlargement (*ex vacuo* hydrocephalus) and internal capsule, and brain stem thinning seem to be the earliest detectable signs of CS on brain imaging (Adachi et al., 2006). Hypomyelination, cerebellar atrophy, and calcification of the dentate nucleus of the cerebellum and the basal ganglia (BG) comprise the most typical pattern recognized in classic and late onset CS (Koob et al., 2010).

These nervous system abnormalities are associated with the neurological symptoms and cognitive impairment that characterize the disease. The severity of cognitive impairment varies in CS. The majority of type I and II CS patients have intellectual disability and severe cognitive deficits (Rapin et al., 2006). Some patients with type III CS have been reported to have mild intellectual disability or even normal intellectual capacities (Nance and Berry, 1992; Rapin et al., 2006). However, even in the late-onset patients, such as the patient described here, dementia in early adulthood is often reported, related to diffuse, and widespread brain cortical atrophy (Laugel, 2013).

Although developmental delay and cognitive impairment have been widely reported, detailed descriptions of the cognitive profiles of patients with CS are scarce. Only two studies (Sugita et al., 1991, 1992) have examined the cognitive profiles of children with type I or II CS using the revised k-form developmental test (Shimazu, 1985), which provides a posture-motor quotient, a cognition-adaptation quotient, and a language-social quotient. The results showed that the neuropsychological impairments are not associated with the white matter changes (Sugita et al., 1992) or the levels of cellular UV-hypersensitivity (Sugita et al., 1991). However, no extensive assessments of the cognitive functioning of CS adults or type III CS patients have been reported.

By contrast, several reports (Neill and Dingwall, 1950; Rapin et al., 2000; Koob et al., 2010), including the original description by Cockayne (1936), have indicated a relative preservation of the social skills of CS patients on the basis of clinical observations. Nonetheless, there are no studies that have formally examined the social cognitive processes in patients with CS.

In sum, there are no reports regarding the cognitive profile of type III CS patients. No studies to date have included a comprehensive neuropsychological and social cognition assessment of these patients. Furthermore, there has been no previous research in individuals with CS that has examined the relationship between the degree of atrophy and patient performance on neuropsychological and social cognition tests.

In this report, we present an atypical patient with type III CS who exceeds the mean life expectancy of individuals with this pathology. Typically, patients with type III CS develop severe dementia after 30 years of age (Laugel, 2013), but the patient described here presents preservation of some cognitive domains. The study of this exceptional case may help to elucidate the fundamental processes that affect the nervous system in premature aging diseases and provide an important source of information for understanding the problems associated with aging.

The main objective of this study was to describe the neuropsychological and social cognition profiles of a patient with type III CS in comparison with a group of healthy controls. Participants underwent a comprehensive assessment that included multiple neuropsychological tests as well as emotion recognition, theory of mind (ToM), and empathy tasks. In addition, we compared the pattern of atrophy observed in the patient to the matched controls and to *ERCC8* gene expression in the brain of a healthy donor. Finally, the results of the neuropsychological and social cognition assessments were interpreted in light of the atrophy pattern of this atypical patient.

## Materials and Methods

### Participants

#### Patient description

Patient F is a 51-year-old male who suffers from the genetic condition type III CS. He has an atypical presentation of the disease characterized by late clinical onset. This patient have the p.Ala160Thr mutation in the CSA/*ERCC8* gene, located on chromosome 5q11. This mutation was previously identified in a Spaniard type III CS patient (Laugel et al., 2010). The sequencing analysis revealed a substitution of G by A at position 478, leading to a change in codon 160, and to the replacement of Ala by Thr in the protein. Specifically, the mutation resulted in an amino acid change at position 160 (GCA ⇒ ACA).

In early childhood, he presented with photosensitivity and speech problems that progressively evolved toward dysarthria, as well as ataxia. Additionally, he showed an early delay in learning, which led him to repeat 4 years of formal education. However, patient F has an average intellectual capacity and completed 11 years of education, a considerable achievement for a patient with such a pervasive developmental disease (Rapin et al., 2006). In adulthood, patient F presents several motor symptoms, including four-limb ataxia that started as lower limb tremor and progressively ascended, leading over a period of 8 years to paraplegia and the incapability to properly feed himself using cutlery. Additionally, patient F temporarily suffers myoclonic movements. Regarding sensory impairments, he presents mixed deafness, for which he requires a hearing aid, and a refractory defect that causes a decrease in visual acuity. However, this visual defect was corrected at the time of assessment and did not affect his performance in visual tasks.

#### Control sample

Two control groups were assessed. First, eight right-handed men (mean age = 50.75 years, SD = 1.04; mean years of formal education = 10.75; SD = 1.04) were recruited for neuropsychological and social cognition assessments (behavior assessment controls, BACs).

A second control group of five healthy men, matched for age and education (mean age = 53.83 years, SD = 10.87; mean years of formal education = 16.5; SD = 4.2), were scanned with a structural MRI to be compared with patient F’s MRI (MRI assessment controls, MACs). Control subjects had no history of neurological or psychiatric conditions. Demographic data were statistically controlled (see socio-demographic and neuropsychological results below). All participants provided written informed consent in agreement with the Declaration of Helsinki, and the Ethics Committee of the Institute of Cognitive Neurology approved this study.

### Instruments and procedures

Patient F was first evaluated via a neurological examination. A blood sample was extracted to assess genetic characterization. Subsequently, the patient and the BACs were assessed with a battery of neuropsychological tests to assess cognitive function and social cognition processes (see below). In separate sessions, the patient and the MACs were scanned with a structural MRI.

#### Neuropsychological assessment

Participants were evaluated with the Wechsler abbreviated scale of intelligence (WASI). This test includes vocabulary and similarities subtests and provides a verbal estimated IQ (Weschler, 1999).

##### General cognitive status

The general participants’ cognitive state was assessed using the Montreal cognitive assessment (MOCA) (Nasreddine et al., 2005). This test was developed for evaluating the general cognitive functions of patients with memory complaints and MCI. It comprises an assessment of short-term memory, visuo-spatial/executive skills (including alternation, phonetic fluency, and abstraction), attention, working memory, language, and orientation. The maximum score is 30 points, and 25 or below indicates impairment.

##### Memory

This cognitive domain was evaluated with the memory capacity test (Buschke, 1984; Grober and Buschke, 1987). This test is intended to evaluate verbal learning through the use of semantic cues that enhance memory encoding. It consists of two lists of 16 words, each distributed over four sheets containing four words belonging to one semantic category. The patient must recall words from the first list, the second list or both, with semantic cues associated with each word’s category (i.e., type of tree, pine). Two scores are calculated: a cued-recall score, which is the sum of all recalled words from each list when semantic cues were provided, and a free-recall score resulting from totaling the correctly evoked words from both lists.

##### Language

Naming and syntax comprehension were evaluated by administering the Boston test (Goodglass et al., 2001). Naming was assessed through the presentation of pictures grouped by categories to determine lexical access. For the assessment of syntactic processing, the subtests of “touch A with B” and “embedded sentences” of the extended version of the Boston Test were used. The patient and controls were also evaluated with the WAIS-III vocabulary subtest (Wechsler, 1997), which assesses the ability to give concise accurate oral definitions of words in increasing order of difficulty.

In addition, we administered two tests of semantic association, the kissing and dancing test (KDT) (Bak and Hodges, 2003) and the pyramids and palm trees test (PPT) (Howard and Patterson, 1992). We used an abbreviated version of the KDT. This test uses 52 triads of images to assess the ability to access semantic representations of verbs. Each triplet is composed of a cue action-picture (e.g., writing) and two semantically related pictures (typing and stirring). Participants are asked to point to the picture that is the most closely related to the cue picture. KDT deficits have already been reported in other movement disorders such as progressive supranuclear palsy (Bak et al., 2006) and early Parkinson’s disease (Ibanez et al., 2013b).

We also used the PPT (Howard and Patterson, 1992), an assessment of semantic associative knowledge of objects. This test consists of 52 triads of images in which participants are asked to select which of two pictures conceptually matches a target picture (i.e., pine tree or palm tree with pyramid).

##### Executive functions

Frontal and executive functions (EF) were evaluated by the administration of the INECO Frontal Screening test (IFS) (Torralva et al., 2009), which assesses eight different domains of frontal lobe function and has previously been used to assess frontal performance in patients with brain damage (Torralva et al., 2009; Gleichgerrcht et al., 2011). The IFS includes the following subtasks: motor programing, conflicting instructions, motor inhibitory control, verbal working memory, backward digit span, spatial working memory, abstraction, and verbal inhibitory control. The maximum possible score on the IFS is 30 points. Finally, participants were evaluated with the WAIS-III similarities subtest (Wechsler, 1997) to evaluate abstract thinking.

#### Social cognition assessment

##### Emotion recognition

Participants were assessed with the emotional morphing test, a facial expression recognition task featuring six basic emotions (happiness, surprise, sadness, fear, anger, and disgust) taken from the pictures of affect series (Ekman and Friesen, 1976), which had been morphed for each prototype emotion and for a neutral state (Young et al., 1997). This procedure involved taking a variable percentage of the shape and texture differences between the two standard images, from 0 (neutral) to 100% (full emotion), in 5% steps (500 ms for each image). The 48 morphed facial stimuli were presented on a computer screen (in a random order) for as long as the patient took to respond by pressing the keyboard. Each participant was asked to respond as soon as they recognized the facial expression and then to identify it from a forced choice list of six options. The accuracy of the emotion recognition and the reaction times were measured.

##### Theory of mind

We employed the Reading the Mind in the Eyes Test (RMET) (Baron-Cohen et al., 1997) to assess the emotional inference of the ToM. The RMET is a computerized and validated test in which 36 images are presented, each showing the region of the face from midway along the nose to just above the eyebrows. The participant is forced to choose which of four words best describes what the person in the picture is thinking or feeling.

##### Empathy

We used an empathy for pain task (EPT) that evaluates empathy for pain in the context of intentional and accidental harm, as well as control situations. The task consists of the successive presentation of 25 animated situations with two persons (Baez et al., 2012, 2013; Decety et al., 2012; Couto et al., 2013). The following three types of situations were depicted: intentional pain, in which one person (passive performer) is in a painful situation caused intentionally by another (active performer), e.g., stepping purposely on someone’s toe (pain caused by other); accidental pain, where one person is in a painful situation accidentally caused by another; and control or neutral situations (e.g., one person receiving a flower given by another). Importantly, the faces of the protagonists were not visible, and there was no emotional reaction visible to the participants. We measured the accuracy of the intentionality identification (the accidental or deliberate nature of the action).

#### Structural brain measures

##### Imaging recordings

Both the patient and the control group were scanned in a 1.5 T Phillips Intera scanner with a standard head coil. A T1-weighted spin echo sequence was used to generate 120 contiguous axial slices (TR = 2300 ms; TE = 13 ms; flip angle = 68°; FOV = rectangular 256 mm; matrix size = 256 × 240 × 120; slice thickness = 1 mm).

### Data analysis

#### Behavioral single-case analysis

To compare the CS patient performance with the control sample (BAC) performance, we used a modified one-tailed *t*-test (Crawford and Howell, 1998; Crawford and Garthwaite, 2002, 2012; Crawford et al., 2009, 2011). This methodology allows the assessment of significance by comparing multiple individuals’ test scores with norms derived from small samples. This modified test is more robust for non-normal distributions, presents low values of type I error, and has already been reported in recent single-case studies (Straube et al., 2010; Couto et al., 2013). Because we are reporting case studies, only values with *p* < 0.05 were considered statistically significant in all comparisons. Effect sizes obtained using the same methods are reported as point estimates (*z*_cc_ as effect size for the modified *t*-test with covariate analysis), as suggested by a previous study (Crawford et al., 2010).

#### Voxel-based morphometry analysis

Images were preprocessed for Voxel-based morphometry (VBM) analysis using DARTEL Toolbox and following previously described procedures (Ashburner and Friston, 2000). Then, modulated, 12 mm full-width half-maximum kernel-smoothed (Good et al., 2001) images were normalized to MNI space and analyzed within general linear models in SPM-8 second level analyses (http://www.fil.ion.ucl.ac.uk/spm/software/spm8). Subsequently, a *t*-test between patient F’s smoothed, normalized, and modulated gray matter images and those of the MAC controls was performed to account for the global atrophy pattern and was corrected by total intracranial volume (TIV). The results of this statistical parametric map were corrected for multiple comparisons with the Bonferroni test (α = 0.05, FEW method), and statistical significance was set at *p* < 0.001.

#### Gene expression and atrophy pattern

To establish a link between the atrophy pattern and the expression of the mutated gene, data from the Allen Human Brain database (Jones et al., 2009) were assessed and compared to the CS patient’s atrophy. The Allen Human Brain database (Jones et al., 2009) is a freely available source in which we performed a search and from which we downloaded data regarding the expression of the *ERCC8* gene in the brain of a healthy donor, matched in age, gender, and ethnicity to our CS patient (donor H0351.1016, 55-year-old Caucasian male). The data obtained from the probes A_23_P58521, A_24_P324986, and CUST_352_PI417507815 include the expression level of this gene and its location on the subject’s brain, detailed in MNI standard coordinates. We computed the spatial overlap of each region within a 5-mm radius of the gene expression and counted the number of these areas that were atrophied in the patient.

## Results

### Neuropsychological assessment

**Figure 1:**
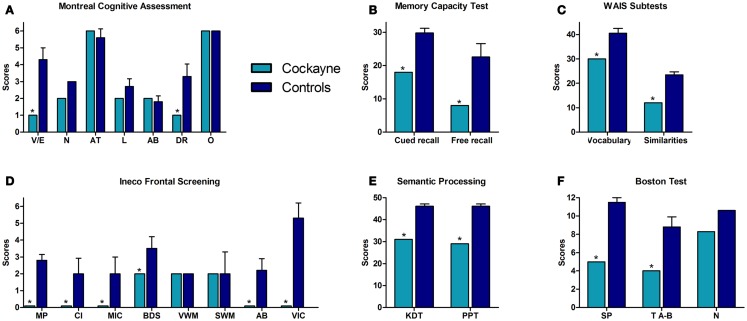
**Performance of the CS patient and controls in neuropsychological tests**. **(A)** Montreal Cognitive Assessment (MOCA). V/E, visuo-spatial/executive skills; N, naming; AT, attention; L, language; AB, abstraction; DR, delayed recall; O, orientation. **(B)** Memory capacity test total scores. **(C)** Vocabulary and similarities raw scores. **(D)** INECO frontal screening subtests. MP, motor programing; CI, conflicting instructions; MIC, motor inhibitory control; BDS, backward digits span; VWM, verbal working memory; SWM, spatial working memory; AB, abstraction; VIC, verbal inhibitory control. **(E)** Semantic processing tasks. KDT, kissing and dancing test; PPT, palm and pyramids test. **(F)** Boston test. SP, syntactic processing; TA-B, touch A with B; N, naming. Asterisk (*) indicates significant differences.

Patient F showed a decreased estimative IQ when compared to matched controls (*t* = −6.64, *p* = 0.0001, *z*_cc_ = −7.04). However, he obtained an estimated IQ of 81, considered to be low-average. Figure 1 summarizes his performance in the different cognitive tasks, compared against controls. See also Table 1A.

**Table 1:** **(A) Estimative intellectual level and general cognitive state, (B) memory capacity test, (C) language, and (D) executive functions**.

Cockayne					Controls
	Score	*t*	*p*	*z*_cc_	
**A**					
IQ total (WASI)	81	−6.64	0.0001*	−7.04	*M* = 96.25; SD = 2.165 (95–100)
MOCA total score	20	−5.903	0.0003*	−6.26	*M* = 27; SD = 1.118 (26–29)
Executive functions	1	−4.572	0.001*	−4.84	*M* = 4.375; SD = 0.696 (3–5)
Attention	6	0.73	0.24	0.77	*M* = 5.625; SD = 0.484 (5–6)
Language	2	−1.633	0.07	−1.732	*M* = 2.75; SD = 0.433 (2–3)
Abstraction	2	0.35	0.36	0.37	*M* = 1.875; SD = 0.331 (1–2)
Delayed recall	1	−3.21	0.007*	−3.41	*M* = 3.375; SD = 0.696 (2–4)
Naming	2	NA	NA	NA	*M* = 3; SD = 0(0)
Orientation	6	NA	NA	NA	*M* = 6; SD = 0(6)
**B**					
Total free-recall	8	−3.71	0.003*	−3.93	*M* = 23; SD = 3.808 (18–29)
Cued-recall
List 1	10	−6.19	0.0002*	−6.56	*M* = 15.125; SD = 0.781 (14–16)
List 2	12	−8.165	0.00004*	−8.661	*M* = 15; SD = 0 (15–15)
List 1 and list 2	18	−8.214	0.00004*	−8.712	*M* = 29.375; SD = 0.696 (28–30)
Free-recall
Total score	8	−3.71	0.003*	−3.93	*M* = 22.62; SD = 4.0 (18–29)
**C**					
“Touch A with B”	5	−12.25	*p* < 0.001*	−13	*M* = 11.5; SD = 0.5 (11–12)
Embedded sentences	4	−7.66	*p* < 0.001*	−8.13	*M* = 8.875; SD = 0.599 (8–10)
Naming	8.33	−1.14	0.14	−1.21	*M* = 10.66; SD = 1.92 (8–12)
Vocabulary (WAIS)	30	−5.36	0.0005*	−5.69	*M* = 40.625; SD = 1.867 (39–44)
Pyramids (PPT)	29	−9.88	0.00001*	−10.48	*M* = 45.625; SD = 1.576 (43–48)
Kissing and dancing (KDT)	31	−13.53	0.001*	−14.36	*M* = 46.125; SD = 1.053 (45–48)
**D**					
INECO frontal screening total	6	−4.83	0.0009*	−5.12	*M* = 22; SD = 3.122 (17–27)
IFS motor programing	0	−8.19	0.00004*	−8.69	*M* = 2.875; SD = 0.331 (2–3)
Conflicting instructions	0	−2.17	0.03*	−2.3	*M* = 2; SD = 0.866 (0–3)
Motor inhibitory control	0	−1.88	0.05*	−2	*M* = 2; SD = 1 (0–3)
Backward digit span	2	−2	0.04*	−2.12	*M* = 3.5; SD = 0.707 (2–4)
Verbal working memory	2	0	1	0	*M* = 2; SD = 0 (2)
Spatial working memory	2	0	0.5	0	*M* = 2; SD = 1.225 (0–3)
Abstraction	0	−7.66	0*	−8.12	*M* = 2.25; SD = 0.661 (1–3)
Verbal inhibitory control	0	−5.91	0.0003*	−6.27	*M* = 5.375; SD = 0.857 (4–6)
Similarities (WAIS)	12	−9.76	0	−10.35	*M* = 23.5; SD = 1.118 (22–25)

**M*, mean; SD, standard deviation, range in parentheses*.

*Significant differences from controls.

*NA, not affordable through statistics*.

#### General cognitive state

In the MOCA, the patient showed a significantly lower total score (*t* = −5.903, *p* = 0.0003, *z*_cc_ = −6.26). Specifically, he failed in executive functions (*t* = −4.572, *p* = 0.001, *z*_cc_ = −4.84) as well as in delayed recall (*t* = −3.21, *p* = 0.007, *z*_cc_ = −3.41). However, no significant differences between the patient and controls were found in naming (patient: mean = 2; controls: mean = 3, SD = 0.0), attention (*t* = 0.73, *p* = 0.24, *z*_cc_ = 0.77), language (*t* = −1.633, *p* = 0.07, *z*_cc_ = −1.732), abstraction (*t* = 0.35, *p* = 0.36, *z*_cc_ = 0.37) or orientation (patient: mean = 6; controls: mean = 6, SD = 0.0). See Table 1A.

#### Memory

Regarding verbal learning, patient F was found to be impaired in the cued-recall scores of the memory capacity test (list 1: *t* = −6.19, *p* = 0.0002, *z*_cc_ = −6.56; list 2: *t* = −8.165, *p* = 0.00004, *z*_cc_ = −8.661; list 1 and 2: *t* = −8.214, *p* = 0.00004, *z*_cc_ = −8.712) and in the total free-recall test (*t* = −3.71, *p* = 0.003, *z*_cc_ = −3.93; 0–30 min). See Table 1B.

#### Language

Regarding syntactic processing, patient F showed a significantly lower score than controls in syntactic processing (*t* = −7.66, *p* = 0.001, *z*_cc_ = −8.13) and in the “touch A with B” task (*t* = −12.25, *p* = 0.001, *z*_cc_ = −13). In the naming task, no significant difference was observed between the patient and controls (*t* = −1.14, *p* = 0.14, *z*_cc_ = −1.21). Furthermore, significantly lower values were observed for patient F in the vocabulary subtest of the WAIS (*t* = −5.36, *p* = 0.0005, *z*_cc_ = −5.69). Moreover, compared to controls, the semantic access of verbs was impaired in patient F as measured by the KDT (*t* = −13.53, *p* = 0.001, *z*_cc_ = −14.36) and the PPT (*t* = −9.88, *p* = 0.001, *z*_cc_ = −10.48). See Table 1C.

#### Executive functions

In the IFS, patient F showed impairment on the total score (*t* = −4.83, *p* = 0.0009, *z*_cc_ = −5.12) and specifically scored lower on motor programing (*t* = −8.19, *p* = 0.00004, *z*_cc_ = −8.69), conflicting instructions (*t* = −2.17, *p* = 0.03, *z*_cc_ = −2.3), motor inhibitory control (*t* = −1.88, *p* = 0.05, *z*_cc_ = −2), backward digit span (*t* = −2, *p* = 0.04, *z*_cc_ = −2.12), verbal inhibitory control (*t* = −5.91, *p* = 0.0003, *z*_cc_ = −6.27), and abstraction (*t* = −7.66, *p* = 0.001, *z*_cc_ = −8.12). However, no differences were observed between F and controls on spatial working memory (*t* = 0, *p* = 0.50, *z*_cc_ = 0) and verbal working memory (*t* = 0, *p* = 1, *z*_cc_ = 0; F’s score = 2 and all controls scored equally). No differences were observed between the patient and controls for the similarities subtest (*t* = −9.76, *p* = 0, *z*_cc_ = −10.35). See Table 1D.

In summary, the neuropsychological assessment revealed that patient F had preserved orientation and attention. However, in the memory test, he showed impairments in both cued and free-recall. Language tests showed preserved naming and repetition. However, syntactic processing and semantic association were affected. Regarding EF, the patient exhibited a normal performance in the IFS subtests of verbal and spatial working memory and in similarities tasks, whereas his motor programing, inhibitory control, and abstraction were impaired. His performance in backward digits span was also impaired.

### Social cognition assessment

**Figure 2:**
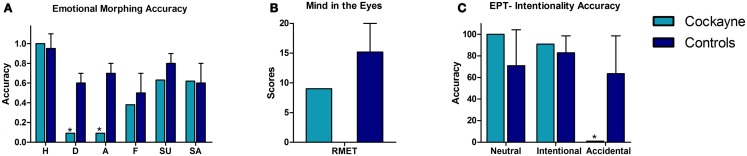
**Performance of the CS patient and controls in social cognition tasks**. **(A)** Emotional morphing (accuracy per category). H, happiness; D, disgust; A, anger; F, fear; SU, surprise; SA, sadness. **(B)** Reading the mind in the eyes total score. **(C)** EPT, Empathy for pain task; intentionality accuracy. Asterisk (*) indicates significant differences.

Figure 2 shows the performance of the CS patient and controls in the social cognition tasks. See also Table 2.

**Table 2:** **Social cognition tasks**.

Cockayne					Controls
	Score	*t*	*p*	*z*_cc_	
**EMOTIONAL MORPHING TEST**					
Happiness	1	0.72	0.24	0.76	*M* = 0.906; SD = 0.136 (0.63–1)
Disgust	0	−3.56	0.004*	−3.77	*M* = 0.657; SD = 0.174 (0.5–0.88)
Anger	0	−5.39	0.0005*	−5.72	*M* = 0.782; SD = 0.137 (0.63–1)
Fear	0.38	−0.52	0.3	−0.55	*M* = 0.531; SD = 0.271 (0.13–1)
Sadness	0.63	−1.04	0.16	−1.1	*M* = 0.813; SD = 0.165 (0.63–1)
Surprise	0.38	−0.87	0.2	−0.92	*M* = 0.625; SD = 0.265 (0.13–1)
*Reading mind in the eyes*	9	−1.3	0.11	−1.38	*M* = 15.25; SD = 4.521 (10–20)
**EMPATHY FOR PAIN**					
Accuracy neutral	3	0.89	0.2	0.94	*M* = 2.13; SD = 0.93 (0–3)
Accuracy intentional pain	10	0.51	0.31	0.54	*M* = 9.125; SD = 1.615 (7–11)
Accuracy accidental pain	0	−1.83	0.05^#^	−1.94	*M* = 7; SD = 3.606 (0–11)

*Significant differences from controls (p < 0.01).

*^#^Significant differences from controls (p < 0.05)*.

#### Emotion recognition

In the emotional morphing test, patient F showed significant differences in recognizing the basic emotions of disgust (*t* = −3.56, *p* = 0.004, *z*_cc_ = −3.77) and anger (*t* = 0.72, *p* = 0.001, *z*_cc_ = −5.72). Nevertheless, he showed spared recognition of happiness (*t* = −3.56, *p* = 0.24, *z*_cc_ = 0.76), fear (*t* = −0.52, *p* = 0.30, *z*_cc_ = −0.55), sadness (*t* = −1.04, *p* = 0.16, *z*_cc_ = −1.1), and surprise (*t* = −0.87, *p* = 0.20, *z*_cc_ = −0.92).

#### Theory of mind

Regarding ToM, patient F showed no deficits compared to controls in inferring the mental and emotional states in the RMET (*t* = −1.3, *p* = 0.11, *z*_cc_ = −1.38).

#### Empathy

In the EPT, patient F showed no significant differences in the categorization of neutral situations (*t* = 0.89, *p* = 0.20, *z*_cc_ = 0.94) and intentional pain situations (*t* = 0.51, *p* = 0.31, *z*_cc_ = 0.54). However, he showed impaired recognition of accidental pain situations (*t* = −1.83, *p* = 0.05, *z*_cc_ = −1.94).

Overall, the social cognition assessment showed that patient F was able to recognize facial emotions of happiness, fear, surprise, and sadness. However, he had difficulties in recognizing negative emotions such as disgust and anger. Furthermore, patient F showed no deficits on inferring the mental and emotional states in the RMET. Finally, in the EPT, he was able to infer the intentionality of intentional and neutral situations, although he had difficulty in identifying the more ambiguous situations (accidental pain situations).

### Imaging results

#### VBM results

##### Global atrophy of patient F compared to healthy brains

**Figure 3:**
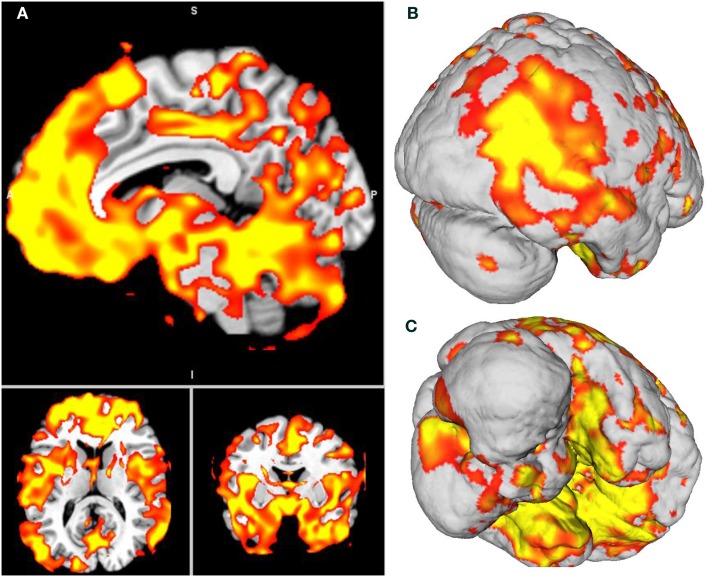
**(A)** Patient F atrophy obtained through voxel-based morphometry analysis. All slices are *p* < 0.001, Bonferroni-corrected (α, 0.05, FWE). **(B,C)** Surface-based 3D reconstruction of patient F’s atrophy plotted over a standard brain in MNI space.

The VBM analysis revealed a pattern of global atrophy in patient F in the frontal structures, bilateral cerebellum, BG and temporal lobe (medial and lateral), and in the occipito-temporal and occipito-parietal regions, as expected and reported in the original descriptions of CS. The affected regions included (all bilaterally) the superior frontal gyrus (SFG); orbito frontal cortex (OFC); rectus gyrus; parahippocampal cortex; and hippocampus; globus pallidus; supplementary motor cortex; inferior temporal gyrus (ITG); medial prefrontal cortex (mPFC) and anterior cingulate (ACC) cortices; superior and mid temporal gyri (STG and MTG); precuneus; insula; fusiform; calcarine sulcus; cuneus; temporal pole; Heschl gyrus; lingual; paracentral lobule; and postcentral gyrus (*p* < 0.001, α = 0.05 FWE corrected). See Table 3 and Figure 3. Table 4 shows the sites of spared gray matter volume in patient F, including the frontal, temporal, and occipito-temporal regions.

**Table 3:** **Brain sites of CS patient significant atrophy**.

Brain region	*x*	*y*	*z*	Cluster *k*	Peak *p* (FWE-cor)	Peak *t*	Peak *z*
Superior frontal gyrus R	18	52.5	3	53	<0.001	531.48	7.24
	15	16.5	54		0.0353	55.57	5.51
	28.5	60	13.5	94	0.0020	98.70	6.00
Orbitofrontal R	13.5	21	−28.5	830	<0.001	400.52	7.05
	43.5	27	−13.5	140	0.0029	91.70	5.94
	36	31.5	−13.5		0.0195	62.62	5.62
Rectus gyrus R	7.5	24	−22.5		<0.001	283.65	6.80
	6	39	−25.5		<0.001	229.31	6.65
Left cerebellum	−3	−37.5	−16.5		<0.001	244.76	6.69
Right cerebellum	9	−43.5	−19.5		<0.001	236.24	6.67
Orbitofrontal L	−19.5	24	−24	330	<0.001	292.72	6.82
	−52.5	28.5	−7.5	127	0.0001	167.03	6.41
Superior frontal gyrus L	−21	36	30	946	<0.001	200.27	6.55
	−16.5	37.5	−24		0.0035	88.26	5.90
	−12	15	55.5		<0.001	73.81	5.76
	−19.5	58.5	7.5		<0.001	169.88	6.42
	−22.5	49.5	22.5		<0.001	187.40	6.50
	−19.5	27	54		0.0063	78.57	5.81
	−18	36	43.5		0.0149	66.04	5.66
Rectus gyrus L	−13.5	34.5	−16.5		0.0338	56.07	5.52
Parahippocampal R	18	−15	−13.5	585	<0.001	265.06	6.75
	36	−21	−22.5		0.0052	81.63	5.84
	22.5	−25.5	−22.5		0.0096	72.14	5.74
Globus pallidus R	15	−3	−10.5		<0.001	215.87	6.60
Hipocampus R	19.5	−12	−34.5		<0.001	106.27	6.06
Supplementary motor L	−4.5	18	58.5	191	<0.001	224.45	6.63
Inferior temporal gyrus R	48	−15	−36	150	<0.001	217.43	6.61
	66	−39	−25.5		0.0320	56.69	5.53
Medial prefrontal L	−7.5	52.5	19.5	1535	<0.001	209.10	6.58
Anterior cingulate L	−1.5	42	12		<0.001	199.00	6.54
Anterior cingulate R	7.5	46.5	16.5		<0.001	191.87	6.51
(Subgenual)	0	3	−12	123	0.0042	85.03	5.87
Precuneus L	−1.5	−72	60	64	0.0001	176.92	6.45
Precuneus R	4.5	−75	52.5		0.0006	125.84	6.19
	6	−82.5	−49.5		0.0061	78.98	5.81
	3	−52.5	37.5	103	0.0022	97.25	5.98
Superior temporal gyrus R	49.5	−58.5	22.5	190	0.0001	173.60	6.44
Mid temporal gyrus R	58.5	−64.5	22.5		0.0020	98.40	5.99
Parahippocampal L	−22.5	−16.5	−31.5	638	0.0001	169.85	6.42
	−13.5	−16.5	−21		0.0018	101.07	6.02
	−12	1.5	−21	54	0.0023	96.28	5.98
Insula L	−33	−3	13.5	332	0.0002	159.72	6.38
Rolandic operculum L	−45	1.5	6		0.0009	115.89	6.13
Hippocampus L	−33	−16.5	−12	336	0.0004	139.73	6.27
	−24	−15	−13.5		0.0013	108.14	6.07
Mid frontal gyrus R	45	49.5	1.5	367	0.0004	136.19	6.25
	39	60	1.5		0.0010	114.33	6.11
	33	30	36	83	0.0008	119.10	6.15
	36	37.5	39		0.0015	104.50	6.04
	31.5	36	46.5		0.0097	72.05	5.73
Orbitofrontal mid R	36	52.5	−15		0.0005	130.55	6.22
	28.5	34.5	−21		0.0213	61.48	5.60
Superior temporal gyrus L	−57	−1.5	−13.5	169	0.0006	127.57	6.20
	−57	18	−9		0.0072	76.33	5.78
	−46.5	0	−4.5		0.0035	88.23	5.90
Mid temporal gyrus L	−61.5	−12	−12		0.0022	96.81	5.98
Inferior temporal gyrus L	−49.5	−10.5	−25.5		0.0035	88.04	5.90
Medial prefrontal R	7.5	−34.5	52.5	304	0.0006	124.39	6.18
Supplementary motor R	6	21	49.5		0.0026	93.76	5.95
	3	6	46.5		0.0049	82.58	5.85
	7.5	−3	48		0.0076	75.54	5.77
Fusiform gyrus R	58.5	−40.5	−30	58	0.0007	121.82	6.16
	42	−27	−19.5	215	0.0010	113.85	6.11
	36	−4.5	−42	107	0.0020	99.10	6.00
	34.5	−12	−34.5		0.0154	65.64	5.66
Mid cingulate R	4.5	−12	49.5	249	0.0009	115.23	6.12
Poscentral gyrus L	−49.5	−7.5	42	93	0.0017	101.97	6.02
Cuneus R	6	−76.5	27	71	0.0022	96.44	5.98
Calcarine sulcus R	1.5	−72	12	100	0.0028	92.18	5.94
Lingual gyrus R	3	−64.5	3		0.0144	66.48	5.67
	15	−49.5	−7.5		0.0079	75.00	5.77
Cerebellum L	−7.5	−57	−12	87	0.0030	90.78	5.93
Insula R	46.5	1.5	−3	70	0.0034	88.89	5.91
Superior temporal pole R	43.5	1.5	−12		0.0095	72.26	5.74
Cerebellar vermis	6	−51	−4.5	131	0.0039	86.39	5.89
Paracentral lobule L	−1.5	−24	57	57	0.0043	84.67	5.87
Heschl gyrus L	−46.5	−12	1.5	72	0.0053	81.18	5.84
	−45	−12	1.5		0.0074	76.01	5.78
	−54	−15	9		0.0173	64.12	5.64
Mid frontal gyrus L	−24	33	49.5	120	0.0053	81.09	5.83

*R, right hemisphere; L, left hemisphere; FWE, family-wise error detection (Bonferroni α = 0.05 corrected results)*.

**Table 4:** **Brain sites of spared gray matter volume in CS patient**.

Region	BA	Coordinates			Cluster *k*	Peak *p* (FWE)	Peak *t*	Peak *z*
		*x*	*y*	*z*	
Gyrus rectus R	11	12	21	−29	28490	0	860.25	6.79
ACC R	24	3	39	7		0.0001	458.35	6.42
Frontal superior R	6	18	53	3		0.0002	438.95	6.39
Lingual R	18	−23	−82	−17	867	0.0002	408.58	6.35
Cerebellum follum 6 L	–	−30	−76	−20		0.0126	147.38	5.69
Fusiform L	36	−38	−25	−15	1256	0.0035	203.33	5.91
	36	−36	−34	−18		0.0064	174.55	5.80
	36	−41	−70	−14		0.0154	140.07	5.66
Mid cingulate R	31	11	−22	43	109	0.0004	357.00	6.27
	31	12	−33	45		0.0357	113.51	5.51
Supramarginal R	40	54	−46	24	43	0.0006	310.93	6.18
Fusiform R	36	39	−24	−18	875	0.0007	300.01	6.16
Cerebellum folli 4–5 R	–	33	−27	−32		0.0026	218.51	5.95
Cerebellum crus 1 R	–	51	−60	−26		0.0044	192.05	5.87
IFG *pars triangularis* L	45	−60	23	10	119	0.0009	284.14	6.12
	45	−63	17	18		0.017	136.66	5.64
	46	−41	27	25	63	0.0592	100.07	5.42
Mid temporal R	21	63	−54	3	201	0.001	277.00	6.11
	21	52	−60	22	359	0.0032	207.44	5.92
	21	54	−48	0		0.0105	154.12	5.72
	21	69	−15	−9	248	0.0401	110.27	5.49
Inferior temporal L	36	−47	−34	−23	52	0.0013	260.25	6.07
Precuneus R	31	5	−81	51	206	0.0015	250.90	6.04
	31	0	−72	57		0.0129	146.48	5.69
	31	5	−61	43	292	0.0356	113.64	5.51
Lingual L	18	−5	−87	−17	134	0.0017	243.22	6.02
Mid cingulate L	31	−5	−43	49	346	0.0026	218.84	5.95
Precuneus L	31	−11	−48	45		0.0643	98.00	5.40
Middle frontal gyrus R	9	33	41	48	360	0.0026	218.00	5.95
	9	54	29	37		0.0028	214.84	5.94
	9	31	30	36		0.0155	139.86	5.65
	9	37	56	24	73	0.0269	121.84	5.56
Insula R	13	40	6	1	984	0.003	211.45	5.93
	13	43	−10	3		0.0292	119.39	5.54
	13	44	−3	−6		0.0538	102.49	5.44
Parahippocampal L	27	−21	−18	−32		0.0065	173.80	5.80
Precentral gyrus R	4	24	−16	57	33	0.0037	200.66	5.90
	4	−48	−4	40	238	0.0041	194.99	5.88
	4	55	12	46	48	0.0389	111.17	5.49
	4	−47	−3	54		0.0913	89.77	5.34
Angular gyrus L	39	−56	−57	24	36	0.0041	194.56	5.88
		−42	−61	31	7	0.0853	91.32	5.35
Angular gyrus R		52	−58	36	55	0.0925	89.50	5.34
Supplementary motor L	6	−5	18	58	257	0.0047	188.50	5.86
	6	−9	24	52		0.0353	113.86	5.51
Superior frontal L	6	−12	15	55		0.0471	105.94	5.46
Middle occipital L	19	−54	−81	6	271	0.0059	178.37	5.82
Brainstem	–	0	−34	−60	24	0.0076	167.28	5.78
	–	9	−43	−69	69	0.0347	114.34	5.51
Superior parietal lobule L	7	−21	−64	64	48	0.0084	162.97	5.76
Postcentral R	5	15	−46	67	55	0.0109	152.74	5.71
Cuneus R	31	9	−75	22	913	0.0182	134.38	5.63
Occipital superior L	18	−14	−79	25		0.0236	125.89	5.58
	18	−24	−75	36		0.024	125.40	5.58
Middle frontal gyrus L	9	−29	8	51	12	0.0186	133.72	5.62
Paracentral lobule L	4	−14	−25	75	39	0.0227	127.15	5.59
	4	−14	−22	66		0.0264	122.41	5.56
Thalamus R	–	17	−25	1		*0.0619*	98.95	5.41
Precentral L	4	−29	−15	52	16	0.0297	118.93	5.54
Superior temporal R	42	57	−33	15	38	0.0323	116.45	5.53
Cerebellum, pyramis	–	−45	−79	−44	53	0.0394	110.80	5.49
Cuneus L	18	−6	−87	16	12	0.0462	106.48	5.46
Superior frontal R	9	23	59	31	30	0.0477	105.59	5.46
Mid temporal L	21	−53	−37	9	13	0.0528	102.94	5.44

*R, right hemisphere; L, left hemisphere; FWE, family-wise error detection (Bonferroni α = 0.05 corrected results); ACC, anterior cingulate cortex; IFG, inferior frontal gyrus*.

#### Gene-atrophy overlapping

**Figure 4:**
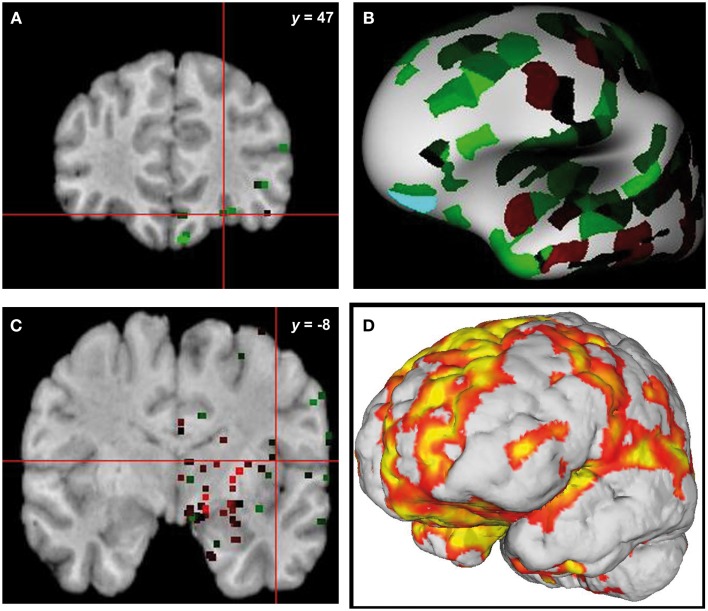
**Overlap of brain sites for *ERCC8* gene expression level in healthy donor and atrophy in CS patient**. **(A,C)** Coronal sections depicting two sites of gene expression/atrophy overlap: orbitofrontal cortex and insular cortex in donor H0351.1016 (Allen Human Brain). **(B)** Three-dimensional surface rendering of the same data as in **(A,C)**. **(D)** Left view of three-dimensional surface rendering of CS patient, showing fronto-insular-temporal and parietal atrophy areas.

From the 80 atrophy peaks reported in Table 3, we found 34 that overlapped with the expression of the *ERCC8* gene in the Allen database (see Figure 4). These coordinates are reported in Table 5 and correspond to the bilateral cerebellum; putamen; orbital and gyrus rectus; superior and mid frontal gyrus; supplementary motor area (SMA); rolandic operculum; mPFC; ACC; mid cingulate; bilateral insula; hippocampus; parahippocampal cortex; superior, mid, and ITG; and left precuneus.

**Table 5:** **Overlap brain sites between the ERCC8 gene expression of Donor H0351.1016 from the Allen Brainmap and CS patient atrophy**.

Donor H0351.1016	MNI Coordinates		
	*x*	*y*	*z*
Superior frontal gyrus R	18	52,5	3
	15	16,5	54
	28,5	60	13,5
Left cerebellum	−3	−37,5	−16,5
Right cerebellum	9	−43,5	−19,5
Orbitofrontal L	−19,5	24	−24
Superior frontal gyrus L	−21	36	30
Rectus gyrus L	−13,5	34,5	−16,5
Supplementary motor L	−4,5	18	58,5
Medial prefrontal L	−7,5	52,5	19,5
Anterior cingulate L	−1,5	42	12
Anterior cingulate R	7,5	46,5	16,5
(subgenual)	0	3	−12
Precuneus L	−1,5	−72	60
Parahippocampal L	−22,5	−16,5	−31,5
Insula L	−33	−3	13,5
Rolandic operculum L	−45	1,5	6
Hippocampus L	−33	−16,5	−12
	−24	−15	−13,5
Mid frontal gyrus R	45	49,5	1,5
	39	60	1,5
	33	30	36
	36	37,5	39
	31,5	36	46,5
Superior temporal gyrus L	−57	−1,5	−13,5
Mid temporal gyrus L	−61,5	−12	−12
Inferior temporal gyrus L	−49,5	−10,5	−25,5
Supplementary motor R	6	21	49,5
	3	6	46,5
	7,5	−3	48
Mid cingulate R	4,5	−12	49,5
Insula R	46,5	1,5	−3

*MNI, Montreal Neurological Instittue, standard space*.

## Discussion

The main goal of this study was to describe the neuroanatomical, neuropsychological, and social cognition profiles of a patient with type III CS in comparison with a group of healthy control subjects.

We also compared the pattern of atrophy observed in the patient to the matched controls and to *ERCC8* gene expression in a healthy brain. Our results showed that patient F had impairments in multiple cognitive domains. Surprisingly, the social cognition assessment revealed that patient F was able to recognize facial emotions, to infer the mental and emotional states, and to accurately identify the intentionality of intentional pain situations. Consistent with previous MRI reports in CS (Adachi et al., 2006; Koob et al., 2010), the VBM analysis revealed a pattern of global cerebral and cerebellar loss of gray matter volume in patient F. These findings were anatomically consistent with the *ERCC8* gene expression pattern in a healthy donor’s brain.

This is the first work to examine the performance of a type III CS patient on a battery of neuropsychological and social cognition tests and to relate his performance with the atrophy pattern. The patient described here represents a rare case of a late-onset type III in whom social cognition skills are better preserved than cognitive functions such as memory, language, and EF. The study of this exceptional case may help to elucidate the processes that affect the brain in premature aging diseases.

### Neuropsychological profile and its relationship to the pattern of atrophy

Patient F had a low estimative IQ compared to controls, but regarding the reference range (Weschler, 2009), his estimative IQ is considered low-average. This result is consistent with previous clinical reports of type III CS patients (Nance and Berry, 1992; Czeizel and Marchalko, 1995; Rapin et al., 2006) showing mild intellectual disability or even normal intellectual capacities. With respect to the general cognitive state, the patient showed a significantly lower MOCA total score than controls, specifically failing in EF and delayed recall.

In the verbal memory test, patient F performed significantly worse than controls on both cued and free-recall. It is worth mentioning that although semantic cues improved his performance, the cued-recall of patient F was still below expectations. These findings suggest deficits in both storage and retrieval processes. The hippocampus, together with adjacent anatomically related cortex, is necessary for the acquisition, temporary storage, and retrieval of explicit memories (Squire et al., 1992). Moreover, neuroimaging studies have shown that retrieval attempt is also associated with activation of the prefrontal cortex (PFC) (Kapur et al., 1995; Rugg et al., 1998). In patient F, the hippocampus and parahippocampal cortex as well as the prefrontal areas SFG, OFC, gyrus rectus, and mPFC were bilaterally affected. Thus, his memory impairments are consistent with the pattern of atrophy revealed by the VBM analysis.

The language assessment revealed that patient F had preserved repetition and naming. Lesion, neuroimaging, and stimulation studies (Topper et al., 1998; Quigg and Fountain, 1999; Buchsbaum et al., 2001; Nozari et al., 2010) have suggested that the left posterior STG participates in phonological aspects of both of these domains of language. More specifically, impaired animal naming correlates with lesions in the anterior inferior temporal lobe, while poor tool naming is associated with damage in the posterior lateral left temporal lobe (Patterson et al., 2007). Moreover, a recent voxel-lesion symptom mapping study (Baldo et al., 2013) showed that picture naming on the Boston test was dependent on a large network of regions, including portions of the left anterior to posterior MTG and STG and underlying white matter, as well as the inferior parietal cortex. Our results showed that portions of the posterior STG and of the MTG are preserved in patient F, which accounts for his normal performance in naming tasks.

Although naming was preserved, patient F showed a low performance in semantic association tasks. We employed two different tasks: the PPT assessed the processing of nouns and objects, and the KDT evaluated the processing of verbs and actions. The CS patient performed significantly lower than controls in both tests. Anatomically, deficits in noun processing have been linked to the anterior temporal cortical regions of the dominant hemisphere, while verb processing has been associated with the anterior frontal regions (Bak and Hodges, 2003). More specifically, tasks involving semantic association of nouns result in focal activation of the anterior inferior temporal lobe, the parahippocampal gyrus, and the inferior occipital cortex (Vandenberghe et al., 1996; Ricci et al., 1999; Butler et al., 2009). In contrast, semantic processing of verbs is linked to the left frontal cortex, particularly to the DLPFC (Perani et al., 1999; Cappa et al., 2002), Brodmann’s areas 44 and 45 (Bak et al., 2001), and the BG involvement (Cardona et al., 2013). The VBM analysis revealed that patient F exhibited considerable atrophy of BG, left anterior ITG and the parahippocampal gyrus as well as the frontal cortex, including the SFG, MFG, and IFG, which are consistent with the noun and verb processing impairments of the patient.

Both naming and semantic association tasks are measures of the semantic memory components (Butler et al., 2009). However, patient F showed an uneven performance in these tests. As mentioned above, he exhibited preserved naming abilities but showed deficits in the PPT and KDT. The discrepancy in the performance between these tasks may be explained by the pattern of atrophy of patient F as well as by the demands of the tests. Semantic association entails knowledge about objects and how the objects relate to one another; it cannot be performed correctly with only reference to the presented stimuli (Bak and Hodges, 2003). This association requires a higher-order knowledge base than simple naming does (Ricci et al., 1999). Thus, semantic association tasks are intrinsically more complex and involve higher cognitive demands than naming tests.

Syntactic processing was also impaired in patient F. Several lines of evidence suggest that syntactic processing is not implemented in a single area but rather that it constitutes an integrated system involving the left DLPFC adjacent to Broca’s area (Indefrey et al., 2001), the anterior portion of the STG (Friederici et al., 2003), the BG (Moro et al., 2001; Friederici et al., 2003; Chan et al., 2013), and the cerebellum (Moro et al., 2001; Murdoch, 2010). In line with previous neuroimaging reports in CS (Koob et al., 2010; Laugel, 2013), the patient described here exhibited significant atrophy of the BG and cerebellum as well as of the anterior portion of the STG. Hence, considering that these fundamental structures for syntactic processing are affected in patient F, deficits in this domain are expected.

With respect to the frontal functions, patient F exhibited severe impairments in motor programing. This finding is consistent with the atrophy of brain areas crucial for this function, such as the caudal portion of the SFG and the supplementary motor cortex. On the other hand, the PFC has systematically been demonstrated to be an important structure for executive functioning (Stuss and Knight, 2002; Fuster, 2008). Nonetheless, recent studies (Dosenbach et al., 2007; Sridharan et al., 2008; Barbey et al., 2012) identified a fronto-parietal network commonly engaged by tasks that require executive processes. This network includes the lateral frontopolar cortex, anterior PFC, cingulate cortex, and inferior and superior parietal lobe. Specifically, verbal inhibitory control and the capacity to respond to conflictive instructions have been associated with the IFG (Stuss and Knight, 2002; Collette et al., 2006). Inhibitory control is also linked to OFC (Collette et al., 2006), while the ACC and the DLPFC are involved in conflict resolution (Stuss and Knight, 2002). The DLPFC also appears to subserve abstract thinking processes (Fuster, 2008). Patient F presented atrophy of these brain regions crucial for executive processes, especially the OFC and DLPFC, that explain his deficits in motor and verbal inhibitory control, in the capacity to respond to conflictive instructions and in abstraction capacity. Concerning working memory subtests, patient F scored significantly lower than controls on the backward digit span, but he performed similarly to controls on verbal and spatial working memory IFS subtests. These uneven results may be related to the sensitivity of the tests. Whereas backward digit span is widely used to sensitively measure working memory (Lezak, 1983), the verbal and spatial working memory subtests are simple, and brief versions of more comprehensive batteries (Hodges, 1994; Weschler, 2009). Hence, backward digit span would be a more adequate measure, and it revealed working memory deficits in patient F. The neural system supporting working memory capacity involves the DLPFC and other interconnected brain areas such as the posterior parietal cortex, the inferior temporal cortex, the cingulate cortex, and the hippocampal formation (Stuss and Knight, 2002). To some extent, most of this neural system was revealed to be damaged in VBM analysis, leading to the expected working memory deficits of patient F.

In sum, our results showed important deficits in memory and EF, which are consistent with the atrophy of the hippocampus and parahippocampal cortex as well as of the PFC. In the language domain, although complex aspects (semantic association and syntactic processing), which are mostly dependent on the PFC and BG, were impaired, other linguistic processes linked to the posterior STG, such as repetition and naming, were preserved.

There are only two studies (Sugita et al., 1991, 1992) that have directly examined the cognitive profile of children with CS, both using the revised k-form developmental test (Shimazu, 1985). Together, these studies assessed three type I CS patients and one patient with type II CS. The results showed that three patients had severe cognitive and motor deficits, and only one of the type I patients evidenced moderate motor impairment and mild cognitive impairment. Although our results are not directly comparable with those of previous studies, they are in line with the clinical descriptions of late-onset type III CS patients (Hashimoto et al., 2008; Laugel, 2013), which report cognitive impairment and early dementia, typically after 30 years of age.

### Social cognition profile and its relationship to the pattern of atrophy

Although several clinical reports (Cockayne, 1936; Neill and Dingwall, 1950; Rapin et al., 2000; Koob et al., 2010) have described a relative preservation of social skills, no studies have formally assessed these processes in patients with CS. The results of the current study showed that patient F was able to identify facial expressions of happiness, surprise, fear, and sadness, although he had difficulties in recognizing disgust and anger. Overall, regions in the temporal lobe, such as the fusiform gyrus, together with a network of structures including the amygdala, OFC, and cingulate cortex, contribute to emotion processing (Adolphs, 2001). Nonetheless, dissociations in the recognition of different facial expressions (e.g., Blair et al., 1999; Lawrence et al., 2007), suggest that different neural systems are specialized, at least in part, for the recognition of particular emotions. For instance, the amygdala appears to link perceptual representations to cognition and behavior on the basis of the emotional value of the stimuli (Adolphs, 2001) and thus appears to be involved in processing the emotional salience of both positive and negative stimuli, with a special role in coding signals of fear (Adolphs, 2001; Britton et al., 2006). The recognition of sadness expressions has been particularly associated with the right inferior and middle temporal gyrus (Blair et al., 1999; Rosen et al., 2006), while disgust recognition has been linked to the insula and the BG (Calder et al., 2000; Adolphs, 2002; Wang et al., 2003; Ibanez et al., 2010; Couto et al., 2013). Regarding anger recognition, the ventral striatum is important for coding human signals of anger (Calder et al., 2004), and atrophy of the vermal region of the cerebellum is associated with impaired recognition of these expressions (Scharmuller et al., 2013). Our results evidenced that patient F had a significant loss of gray matter in the BG, the insula, and the cerebellum, which could explain the selective impairment in anger and disgust recognition. In contrast, the amygdala, part of the fusiform gyrus and parts of the middle and posterior cingulate cortices were fairly preserved, resulting in spared happiness, surprise, fear, and sadness recognition. Furthermore, these results support the findings (Adolphs, 2001; Calder et al., 2004; Lawrence et al., 2007) that particular brain regions are critical nodes of the emotion processing networks and contribute disproportionately (although not exclusively) to the recognition of certain emotions.

Regarding the ToM assessment, we found no differences between patient F and controls in the RMET, which suggests a normal performance in mental state discrimination. Previous studies (Adolphs, 2001; Siegal and Varley, 2002; Saxe and Baron-Cohen, 2006) have identified a widely distributed neural system implicated in ToM. This system includes the amygdala circuit (Siegal and Varley, 2002), the temporo-parietal junction (TPJ), and the mPFC (Adolphs, 2001; Saxe and Baron-Cohen, 2006). There are two possible explanations for the preserved performance of patient F in the RMET. First, neuroimaging studies using this test (Baron-Cohen et al., 1999; Adolphs et al., 2002; Adams et al., 2010) have consistently shown activation of the amygdala, the IFG, and the superior temporal sulcus, structures relatively preserved in patient F. Second, although the RMET is a widely used task, it is a measure of mental state discrimination, it has a direct association with emotion recognition (Ibanez et al., 2013a), and it represents the first stage of attribution of ToM (Ibanez et al., 2013c). This test does not include the second dimension of ToM: inferring the specific content of that mental state (e.g., detecting that someone is happy because he won the lottery) (Baron-Cohen et al., 2001; Fertuck et al., 2009). Consequently, the RMET shares some conceptual overlap with measures of emotion recognition, a domain partially preserved in patient F. Further studies should assess more complex aspects of ToM in patients with CS.

Finally, in the EPT, patient F accurately discriminated the intentionality of neutral and intentional situations, but he exhibited deficits in distinguishing accidental pain situations. Research on empathy for pain has evidenced a neural network, called the pain matrix, that is implicated in the experience of physical pain and is also involved in the perception or even the imagination of another individual in pain (Jackson et al., 2006; Melloni et al., 2013). This neural network includes the SMA, ACC, anterior insula, and amygdala (Singer and Lamm, 2009; Decety et al., 2012). Although several structures of the pain matrix, including the SMA, the ACC, and the insula, were affected in patient F, he only had difficulties in identifying the accidental pain situations. These results are expected because contextual cues are less clear and explicit in these scenarios than in the intentional pain scenarios, making the interpretation of action intentionality less affordable. Moreover, these findings suggest that the integrity of the pain matrix is required for contextual appraisal (Melloni et al., 2013) and for accurately inferring the intentionality of otherwise ambiguous pain situations.

### Implications and future directions

As we mentioned above, our results are not directly comparable to previous studies on CS because the lack of research on adults with type III CS. However, the findings we described are anatomically consistent with the *ERCC8* gene expression level in a healthy donor’s brain. As stated before, mutation of this gene affects DNA repair, which might be causally linked to accelerated aging and neurodegenerative signatures of progeroid syndromes (Borgesius et al., 2011). In particular, neuropathologic changes such as demyelization and selective gray matter atrophy occurring in type III CS (Rapin et al., 2006; Weidenheim et al., 2009) (typical signs of neurodegeneration) would correlate with the specific behavioral impairments and neuropsychological profile of our patient. Moreover, this finding is in accordance with the hypothesis of selective atrophy targeting functionally connected nodes of different large-scale networks in the brain (Seeley et al., 2009).

Interestingly, social cognition domains were better preserved than non-social cognitive processes in patient F, suggesting a dissociation between social and cognitive skills. The current findings are consistent with several clinical reports (Rapin et al., 2006; Weidenheim et al., 2009; Laugel, 2013), including the original description of the syndrome (Cockayne, 1936), in which CS patients are characterized as having normal social skills and engaging, outgoing and friendly personalities, despite their cognitive impairments.

Consistent with our findings, a similar pattern of relative preservation of social cognition skills has been described in patients who suffer from other genetic disorders such as Williams syndrome. Individuals with this disease have high sociability and empathy for others, contrasting with their intellectual disability, long term memory, visuo-spatial abilities, and EF deficits (Meyer-Lindenberg et al., 2006; Rhodes et al., 2010; Haas and Reiss, 2012). Moreover, although both EF and social cognition have been associated with PFC functioning, patient F showed a deep impairment in EF and partial preservation of emotion recognition, ToM, and empathy for pain. Consistent with these findings, previous studies in neurodegenerative diseases (Lough et al., 2001) and in patients with PFC lesions (Sarazin et al., 1998; Fine et al., 2001) have suggested a dissociation of social cognition and EF. Thus, our results, together with previous evidence, support the existence of a partially and functionally independent neural network for social cognition.

In addition, the fact that social cognition abilities are better preserved than other cognitive functions in a patient suffering from a genetic disorder with generalized brain atrophy might be related to several factors that should be further explored. First, humans are an exceedingly social species (Adolphs, 1999; Frith and Frith, 2007; Ibanez and Manes, 2012), and our survival and success depend crucially on our ability to thrive in social situations (Phillips, 2003). Indeed, social abilities and interpersonal skills are ubiquitous among primates and along the human lifespan, making plausible the existence of distinct functionally independent neural networks. Second, it is unknown whether some neural systems are more resilient and plastic than others (Cicchetti, 2013), but considering the crucial importance of social cognition for human survival, it is possible that neural networks supporting these processes have more capacity to respond to intrinsic and extrinsic stimuli by reorganizing their structure, function, and connections. Finally, several studies in healthy subjects (Golomb et al., 1993; MacPherson et al., 2002; Raz et al., 2004) have shown that normal aging mainly affects recent memory and EF, while emotional processing and social behavior remain relatively intact. These findings suggest that social cognition processes seem to be less vulnerable to the effects of aging on the brain.

In conclusion, our study documents the first description of the neuropsychological and social cognition profiles of an adult with type III CS. The results showed memory, language, and EF deficits that contrast with relative preservation of social cognition skills. The cognitive profile of patient F was consistent with his pattern of global cerebral and cerebellar loss of gray matter volume, which in turn was anatomically consistent with the *ERCC8* gene expression level in a healthy donor’s brain.

Cockayne syndrome (as well as other progeroid diseases) is a heritable human disorder with premature aging. These syndromes have been well characterized as clinical entities, and in many instances, the associated genes and causative mutations have been identified (Kudlow et al., 2007). The identification of genes and the study of the cognitive and social processes that are associated with these syndromes, together with the neuropathological examinations of brains of individuals with and without neurodegenerative diseases (Grinberg et al., 2007) are fundamental for the understanding of the molecular mechanisms and symptoms associated with human aging. Further studies on other types of CS and other progeroid syndromes should formally assess cognitive and social functioning in relation to the brain atrophy and the expression of the implicated genes.

## Conflict of Interest Statement

The authors declare that the research was conducted in the absence of any commercial or financial relationships that could be construed as a potential conflict of interest.

## References

[B1] Adachi M. Kawanami T. Ohshima F. Hosoya T. (2006). MR findings of cerebral white matter in Cockayne syndrome. Magn. Reson. Med. Sci. 5, 41–45 10.2463/mrms.5.41 16785726

[B2] Adams R. B. Jr. Rule N. O. Franklin R. G. Jr. Wang E. Stevenson M. T. Yoshikawa S. (2010). Cross-cultural reading the mind in the eyes: an fMRI investigation. J. Cogn. Neurosci. 22, 97–108 10.1162/jocn.2009.21187 19199419

[B3] Adolphs R. (1999). Social cognition and the human brain. Trends Cogn. Sci. 3, 469–479 10.1016/S1364-6613(99)01399-6 10562726

[B4] Adolphs R. (2001). The neurobiology of social cognition. Curr. Opin. Neurobiol. 11, 231–239 10.1016/S0959-4388(00)00202-6 11301245

[B5] Adolphs R. (2002). Neural systems for recognizing emotion. Curr. Opin. Neurobiol. 12, 169–177 10.1016/S0959-4388(02)00301-X 12015233

[B6] Adolphs R. Baron-Cohen S. Tranel D. (2002). Impaired recognition of social emotions following amygdala damage. J. Cogn. Neurosci. 14, 1264–1274 10.1162/089892902760807258 12495531

[B7] Ashburner J. Friston K. J. (2000). Voxel-based morphometry – the methods. Neuroimage 11, 805–821 10.1006/nimg.2000.0582 10860804

[B8] Baez S. Herrera E. Villarin L. Theil D. Gonzalez-Gadea M. L. Gomez P. (2013). Contextual social cognition impairments in schizophrenia and bipolar disorder. PLoS ONE 8:e57664 10.1371/journal.pone.0057664 23520477 PMC3592887

[B9] Baez S. Rattazzi A. Gonzalez-Gadea M. L. Torralva T. Vigliecca N. S. Decety J. (2012). Integrating intention and context: assessing social cognition in adults with Asperger syndrome. Front. Hum. Neurosci. 6:302 10.3389/fnhum.2012.00302 23162450 PMC3492863

[B10] Bak T. H. Hodges J. R. (2003). Kissing and dancing – a test to distinguish the lexical and conceptual contributions to noun/verb and action/object dissociation. Preliminary results in patients with frontotemporal dementia. J. Neurolinguist. 16, 169–181 10.1016/S0911-6044(02)00011-8

[B11] Bak T. H. O’Donovan D. G. Xuereb J. H. Boniface S. Hodges J. R. (2001). Selective impairment of verb processing associated with pathological changes in Brodmann areas 44 and 45 in the motor neurone disease-dementia-aphasia syndrome. Brain 124, 103–120 10.1093/brain/124.1.103 11133791

[B12] Bak T. H. Yancopoulou D. Nestor P. J. Xuereb J. H. Spillantini M. G. Pulvermuller F. (2006). Clinical, imaging and pathological correlates of a hereditary deficit in verb and action processing. Brain 129, 321–332 10.1093/brain/awh701 16330501

[B13] Baldo J. V. Arevalo A. Patterson J. P. Dronkers N. F. (2013). Grey and white matter correlates of picture naming: evidence from a voxel-based lesion analysis of the Boston Naming Test. Cortex 49, 658–667 10.1016/j.cortex.2012.03.001 22482693 PMC3613759

[B14] Barbey A. K. Colom R. Solomon J. Krueger F. Forbes C. Grafman J. (2012). An integrative architecture for general intelligence and executive function revealed by lesion mapping. Brain 135, 1154–1164 10.1093/brain/aws021 22396393 PMC3326251

[B15] Baron-Cohen S. Jolliffe T. Mortimore C. Robertson M. (1997). Another advanced test of theory of mind: evidence from very high functioning adults with autism or asperger syndrome. J. Child Psychol. Psychiatry 38, 813–822 10.1111/j.1469-7610.1997.tb01599.x 9363580

[B16] Baron-Cohen S. Ring H. A. Wheelwright S. Bullmore E. T. Brammer M. J. Simmons A. (1999). Social intelligence in the normal and autistic brain: an fMRI study. Eur. J. Neurosci. 11, 1891–1898 10.1046/j.1460-9568.1999.00621.x 10336657

[B17] Baron-Cohen S. Wheelwright S. Hill J. Raste Y. Plumb I. (2001). The “Reading the Mind in the Eyes” Test revised version: a study with normal adults, and adults with Asperger syndrome or high-functioning autism. J. Child Psychol. Psychiatry 42, 241–251 10.1111/1469-7610.00715 11280420

[B18] Blair R. J. Morris J. S. Frith C. D. Perrett D. I. Dolan R. J. (1999). Dissociable neural responses to facial expressions of sadness and anger. Brain 122(Pt 5), 883–893 10.1093/brain/122.5.883 10355673

[B19] Borgesius N. Z. De Waard M. C. Van Der Pluijm I. Omrani A. Zondag G. C. Van Der Horst G. T. (2011). Accelerated age-related cognitive decline and neurodegeneration, caused by deficient DNA repair. J. Neurosci. 31, 12543–12553 10.1523/JNEUROSCI.1589-11.2011 21880916 PMC6703271

[B20] Britton J. C. Phan K. L. Taylor S. F. Welsh R. C. Berridge K. C. Liberzon I. (2006). Neural correlates of social and nonsocial emotions: an fMRI study. Neuroimage 31, 397–409 10.1016/j.neuroimage.2005.11.027 16414281

[B21] Buchsbaum B. Hickok G. Humphries C. (2001). Role of left posterior superior temporal gyrus in phonological processing for speech perception and production. Cogn. Sci. 25, 663–678 10.1207/s15516709cog2505_216413796

[B22] Buschke H. (1984). Cued recall in amnesia. J. Clin. Neuropsychol. 6, 433–440 10.1080/01688638408401233 6501581

[B23] Butler C. R. Brambati S. M. Miller B. L. Gorno-Tempini M. L. (2009). The neural correlates of verbal and nonverbal semantic processing deficits in neurodegenerative disease. Cogn. Behav. Neurol. 22, 73–80 10.1097/WNN.0b013e318197925d 19506422 PMC2754058

[B24] Calder A. J. Keane J. Lawrence A. D. Manes F. (2004). Impaired recognition of anger following damage to the ventral striatum. Brain 127, 1958–1969 10.1093/brain/awh214 15289264

[B25] Calder A. J. Keane J. Manes F. Antoun N. Young A. W. (2000). Impaired recognition and experience of disgust following brain injury. Nat. Neurosci. 3, 1077–1078 10.1038/80586 11036262

[B26] Cappa S. F. Sandrini M. Rossini P. M. Sosta K. Miniussi C. (2002). The role of the left frontal lobe in action naming: rTMS evidence. Neurology 59, 720–723 10.1212/WNL.59.5.720 12221163

[B27] Cardona J. F. Gershanik O. Gelormini-Lezama C. Houck A. L. Cardona S. Kargieman L. (2013). Action-verb processing in Parkinson’s disease: new pathways for motor-language coupling. Brain Struct. Funct. 218, 1355–1373 10.1007/s00429-013-0510-1 23412746

[B28] Chan S. H. Ryan L. Bever T. G. (2013). Role of the striatum in language: syntactic and conceptual sequencing. Brain Lang. 125, 283–294 10.1016/j.bandl.2011.11.005 22200490

[B29] Cicchetti D. (2013). Annual research review: resilient functioning in maltreated children – past, present, and future perspectives. J. Child Psychol. Psychiatry 54, 402–422 10.1111/j.1469-7610.2012.02608.x 22928717 PMC3514621

[B30] Cockayne E. (1936). Dwarfism with retinal atrophy and deafness. Arch. Dis. Child. 11, 1–8 10.1136/adc.11.61.1 21032019 PMC1975412

[B31] Collette F. Hogge M. Salmon E. Van Der Linden M. (2006). Exploration of the neural substrates of executive functioning by functional neuroimaging. Neuroscience 139, 209–221 10.1016/j.neuroscience.2005.05.035 16324796

[B32] Couto B. Sedeno L. Sposato L. A. Sigman M. Riccio P. M. Salles A. (2013). Insular networks for emotional processing and social cognition: comparison of two case reports with either cortical or subcortical involvement. Cortex 49, 1420–1434 10.1016/j.cortex.2012.08.006 23036522

[B33] Crawford J. R. Garthwaite P. H. (2002). Investigation of the single case in neuropsychology: confidence limits on the abnormality of test scores and test score differences. Neuropsychologia 40, 1196–1208 10.1016/S0028-3932(01)00224-X 11931923

[B34] Crawford J. R. Garthwaite P. H. (2012). Single-case research in neuropsychology: a comparison of five forms of t-test for comparing a case to controls. Cortex 48, 1009–1016 10.1016/j.cortex.2011.06.021 21843884

[B35] Crawford J. R. Garthwaite P. H. Howell D. C. (2009). On comparing a single case with a control sample: an alternative perspective. Neuropsychologia 47, 2690–2695 10.1016/j.neuropsychologia.2009.04.011 19383506

[B36] Crawford J. R. Garthwaite P. H. Porter S. (2010). Point and interval estimates of effect sizes for the case-controls design in neuropsychology: rationale, methods, implementations, and proposed reporting standards. Cogn. Neuropsychol 27, 245–260 10.1080/02643294.2010.513967 20936548

[B37] Crawford J. R. Garthwaite P. H. Ryan K. (2011). Comparing a single case to a control sample: testing for neuropsychological deficits and dissociations in the presence of covariates. Cortex 47, 1166–1178 10.1016/j.cortex.2011.02.017 21458788

[B38] Crawford J. R. Howell D. C. (1998). Comparing an individual’s test score against norms derived from small samples. Clin. Neuropsychol. 12, 482–486 10.1076/clin.12.4.482.7241

[B39] Czeizel A. E. Marchalko M. (1995). Cockayne syndrome type III with high intelligence. Clin. Genet. 48, 331–333 10.1111/j.1399-0004.1995.tb04121.x 8835332

[B40] Decety J. Michalska K. J. Kinzler K. D. (2012). The contribution of emotion and cognition to moral sensitivity: a neurodevelopmental study. Cereb. Cortex 22, 209–220 10.1093/cercor/bhr111 21616985

[B41] Dosenbach N. U. Fair D. A. Miezin F. M. Cohen A. L. Wenger K. K. Dosenbach R. A. (2007). Distinct brain networks for adaptive and stable task control in humans. Proc. Natl. Acad. Sci. U.S.A. 104, 11073–11078 10.1073/pnas.0704320104 17576922 PMC1904171

[B42] Ekman P. Friesen E. (1976). Pictures of Facial Affects. Palo Alto, CA: Consulting Psychologists Press

[B43] Fertuck E. A. Jekal A. Song I. Wyman B. Morris M. C. Wilson S. T. (2009). Enhanced ‘Reading the Mind in the Eyes’ in borderline personality disorder compared to healthy controls. Psychol. Med. 39, 1979–1988 10.1017/S003329170900600X 19460187 PMC3427787

[B44] Fine C. Lumsden J. Blair R. J. (2001). Dissociation between ‘theory of mind’ and executive functions in a patient with early left amygdala damage. Brain 124, 287–298 10.1093/brain/124.2.287 11157556

[B45] Friederici A. D. Ruschemeyer S. A. Hahne A. Fiebach C. J. (2003). The role of left inferior frontal and superior temporal cortex in sentence comprehension: localizing syntactic and semantic processes. Cereb. Cortex 13, 170–177 10.1093/cercor/13.2.170 12507948

[B46] Frith C. D. Frith U. (2007). Social cognition in humans. Curr. Biol. 17, R724–R732 10.1016/j.cub.2007.05.068 17714666

[B47] Fuster J. (2008). The Prefrontal Cortex. San Diego: Elsevier

[B48] Ghai S. J. Shago M. Shroff M. Yoon G. (2011). Cockayne syndrome caused by paternally inherited 5 Mb deletion of 10q11.2 and a frameshift mutation of ERCC6. Eur. J. Med. Genet. 54, 272–276 10.1016/j.ejmg.2011.02.008 21376145

[B49] Gleichgerrcht E. Roca M. Manes F. Torralva T. (2011). Comparing the clinical usefulness of the Institute of Cognitive Neurology (INECO) Frontal Screening (IFS) and the Frontal Assessment Battery (FAB) in frontotemporal dementia. J. Clin. Exp. Neuropsychol. 33, 997–1004 10.1080/13803395.2011.589375 21923634

[B50] Golomb J. De Leon M. J. Kluger A. George A. E. Tarshish C. Ferris S. H. (1993). Hippocampal atrophy in normal aging. An association with recent memory impairment. Arch. Neurol. 50, 967–973 10.1001/archneur.1993.00540090066012 8363451

[B51] Good C. D. Johnsrude I. S. Ashburner J. Henson R. N. Friston K. J. Frackowiak R. S. (2001). A voxel-based morphometric study of ageing in 465 normal adult human brains. Neuroimage 14, 21–36 10.1006/nimg.2001.0857 11525331

[B52] Goodglass H. Kaplan E. Barresi B. (2001). Boston Diagnostic Aphasia Examination. Austin, TX: Pro-Ed

[B53] Grinberg L. T. Ferretti R. E. Farfel J. M. Leite R. Pasqualucci C. A. Rosemberg S. (2007). Brain bank of the Brazilian aging brain study group – a milestone reached and more than 1,600 collected brains. Cell Tissue Bank. 8, 151–162 10.1007/s10561-006-9022-z 17075689

[B54] Grober E. Buschke H. (1987). Genuine memory deficits in dementia. Dev. Neuropsychol. 3, 13–36 10.1080/87565648709540361

[B55] Haas B. W. Reiss A. L. (2012). Social brain development in williams syndrome: the current status and directions for future research. Front. Psychol. 3:186 10.3389/fpsyg.2012.00186 22701108 PMC3370330

[B56] Hanawalt P. C. (2000). DNA repair. the bases for Cockayne syndrome. Nature 405, 415–416 10.1038/35013197 10839526

[B57] Hashimoto S. Suga T. Kudo E. Ihn H. Uchino M. Tateishi S. (2008). Adult-onset neurological degeneration in a patient with Cockayne syndrome and a null mutation in the CSB gene. J. Invest. Dermatol. 128, 1597–1599 10.1038/sj.jid.5701210 18185538

[B58] Hodges J. (1994). Cognitive Assessment for Clinicians. Oxford: Oxford University Press

[B59] Howard D. Patterson K. (1992). Pyramids and Palm Trees: A Test of Semantic Access from Pictures and Words. Bury St. Edmuds: Thames Valley Test Company

[B60] Ibanez A. Aguado J. Baez S. Huepe D. Lopez V. Ortega R. (2013a). From neural signatures of emotional modulation to social cognition: individual differences in healthy volunteers and psychiatric participants. Soc. Cogn. Affect. Neurosci. 10.1093/scan/nst067 23685775 PMC4090956

[B61] Ibanez A. Cardona J. F. Dos Santos Y. V. Blenkmann A. Aravena P. Roca M. (2013b). Motor-language coupling: direct evidence from early Parkinson’s disease and intracranial cortical recordings. Cortex 49, 968–984 10.1016/j.cortex.2012.02.014 22482695

[B62] Ibanez A. Huepe D. Gempp R. Gutiérrez V. Rivera-Rei A. Toledo M. I. (2013c). Empathy, sex and fluid intelligence as predictors of theory of mind. Pers. Individ. Dif. 54, 616–621 10.1016/j.paid.2012.11.022

[B63] Ibanez A. Gleichgerrcht E. Manes F. (2010). Clinical effects of insular damage in humans. Brain Struct. Funct. 214, 397–410 10.1007/s00429-010-0256-y 20512375

[B64] Ibanez A. Manes F. (2012). Contextual social cognition and the behavioral variant of frontotemporal dementia. Neurology 78, 1354–1362 10.1212/WNL.0b013e3182518375 22529204 PMC3335455

[B65] Indefrey P. Hagoort P. Herzog H. Seitz R. J. Brown C. M. (2001). Syntactic processing in left prefrontal cortex is independent of lexical meaning. Neuroimage 14, 546–555 10.1006/nimg.2001.0867 11506529

[B66] Jackson P. L. Brunet E. Meltzoff A. N. Decety J. (2006). Empathy examined through the neural mechanisms involved in imagining how I feel versus how you feel pain. Neuropsychologia 44, 752–761 10.1016/j.neuropsychologia.2005.07.015 16140345

[B67] Jones A. R. Overly C. C. Sunkin S. M. (2009). The Allen Brain Atlas: 5 years and beyond. Nat. Rev. Neurosci. 10, 821–828 10.1038/nrn2722 19826436

[B68] Kapur S. Craik F. I. Jones C. Brown G. M. Houle S. Tulving E. (1995). Functional role of the prefrontal cortex in retrieval of memories: a PET study. Neuroreport 6, 1880–1884 10.1097/00001756-199510020-00014 8547589

[B69] Koob M. Laugel V. Durand M. Fothergill H. Dalloz C. Sauvanaud F. (2010). Neuroimaging in Cockayne syndrome. AJNR Am. J. Neuroradiol. 31, 1623–1630 10.3174/ajnr.A2135 20522568 PMC7964976

[B70] Kudlow B. A. Kennedy B. K. Monnat R. J. Jr. (2007). Werner and Hutchinson-Gilford progeria syndromes: mechanistic basis of human progeroid diseases. Nat. Rev. Mol. Cell Biol. 8, 394–404 10.1038/nrm2161 17450177

[B71] Laugel V. (2013). Cockayne syndrome: the expanding clinical and mutational spectrum. Mech. Ageing Dev. 134, 161–170 10.1016/j.mad.2013.02.006 23428416

[B72] Laugel V. Dalloz C. Durand M. Sauvanaud F. Kristensen U. Vincent M. C. (2010). Mutation update for the CSB/ERCC6 and CSA/ERCC8 genes involved in Cockayne syndrome. Hum. Mutat. 31, 113–126 10.1002/humu.21154 19894250

[B73] Lawrence A. D. Goerendt I. K. Brooks D. J. (2007). Impaired recognition of facial expressions of anger in Parkinson’s disease patients acutely withdrawn from dopamine replacement therapy. Neuropsychologia 45, 65–74 10.1016/j.neuropsychologia.2006.04.016 16780901

[B74] Lezak M. (1983). Neuropsychological Assessment. New York: Oxford University Press

[B75] Lough S. Gregory C. Hodges J. R. (2001). Dissociation of social cognition and executive function in frontal variant frontotemporal dementia. Neurocase 7, 123–130 10.1093/neucas/7.2.123 11320160

[B76] MacPherson S. E. Phillips L. H. Della Sala S. (2002). Age, executive function, and social decision making: a dorsolateral prefrontal theory of cognitive aging. Psychol. Aging 17, 598–609 10.1037/0882-7974.17.4.598 12507357

[B77] Melloni M. Lopez V. Ibanez A. (2013). Empathy and contextual social cognition. Cogn. Affect. Behav. Neurosci. 10.3758/s13415-013-0205-3 23955101

[B78] Meyer-Lindenberg A. Mervis C. B. Berman K. F. (2006). Neural mechanisms in Williams syndrome: a unique window to genetic influences on cognition and behaviour. Nat. Rev. Neurosci. 7, 380–393 10.1038/nrn1906 16760918

[B79] Moro A. Tettamanti M. Perani D. Donati C. Cappa S. F. Fazio F. (2001). Syntax and the brain: disentangling grammar by selective anomalies. Neuroimage 13, 110–118 10.1006/nimg.2000.0668 11133314

[B80] Murdoch B. E. (2010). The cerebellum and language: historical perspective and review. Cortex 46, 858–868 10.1016/j.cortex.2009.07.018 19828143

[B81] Nance M. A. Berry S. A. (1992). Cockayne syndrome: review of 140 cases. Am. J. Med. Genet. 42, 68–84 10.1002/ajmg.1320420115 1308368

[B82] Nasreddine Z. S. Phillips N. A. Bedirian V. Charbonneau S. Whitehead V. Collin I. (2005). The Montreal Cognitive Assessment, MoCA: a brief screening tool for mild cognitive impairment. J. Am. Geriatr. Soc. 53, 695–699 10.1111/j.1532-5415.2005.53221.x 15817019

[B83] Natale V. (2011). A comprehensive description of the severity groups in Cockayne syndrome. Am. J. Med. Genet. A 155A, 1081–1095 10.1002/ajmg.a.33933 21480477

[B84] Neill C. A. Dingwall M. M. (1950). A syndrome resembling progeria: a review of two cases. Arch. Dis. Child. 25, 213–223 10.1136/adc.25.123.213 14783428 PMC1988295

[B85] Nozari N. Kittredge A. K. Dell G. S. Schwartz M. F. (2010). Naming and repetition in aphasia: steps, routes, and frequency effects. J. Mem. Lang. 63, 541–559 10.1016/j.jml.2010.08.001 21076661 PMC2976549

[B86] Patterson K. Nestor P. J. Rogers T. T. (2007). Where do you know what you know? The representation of semantic knowledge in the human brain. Nat. Rev. Neurosci. 8, 976–987 10.1038/nrn2277 18026167

[B87] Perani D. Cappa S. F. Schnur T. Tettamanti M. Collina S. Rosa M. M. (1999). The neural correlates of verb and noun processing. a PET study. Brain 122(Pt 12), 2337–2344 10.1093/brain/122.12.2337 10581226

[B88] Phillips M. L. (2003). Understanding the neurobiology of emotion perception: implications for psychiatry. Br. J. Psychiatry 182, 190–192 10.1192/bjp.02.185 12611778

[B89] Quigg M. Fountain N. B. (1999). Conduction aphasia elicited by stimulation of the left posterior superior temporal gyrus. J. Neurol. Neurosurg. Psychiatry 66, 393–396 10084542 10.1136/jnnp.66.3.393 PMC1736266

[B90] Rapin I. Lindenbaum Y. Dickson D. W. Kraemer K. H. Robbins J. H. (2000). Cockayne syndrome and xeroderma pigmentosum: DNA repair disorders with overlaps and paradoxes. Neurology 55, 1442–1449 10.1212/WNL.55.10.1442 11185579 PMC4459578

[B91] Rapin I. Weidenheim K. Lindenbaum Y. Rosenbaum P. Merchant S. N. Krishna S. (2006). Cockayne syndrome in adults: review with clinical and pathologic study of a new case. J. Child Neurol. 21, 991–1006 10.1177/08830738060210110101 17092472 PMC2772653

[B92] Raz N. Rodrigue K. M. Head D. Kennedy K. M. Acker J. D. (2004). Differential aging of the medial temporal lobe: a study of a five-year change. Neurology 62, 433–438 10.1212/01.WNL.0000106466.09835.46 14872026

[B93] Rhodes S. M. Riby D. M. Park J. Fraser E. Campbell L. E. (2010). Executive neuropsychological functioning in individuals with Williams syndrome. Neuropsychologia 48, 1216–1226 10.1016/j.neuropsychologia.2009.12.021 20026085

[B94] Ricci P. T. Zelkowicz B. J. Nebes R. D. Meltzer C. C. Mintun M. A. Becker J. T. (1999). Functional neuroanatomy of semantic memory: recognition of semantic associations. Neuroimage 9, 88–96 10.1006/nimg.1998.0386 9918730

[B95] Rosen H. J. Wilson M. R. Schauer G. F. Allison S. Gorno-Tempini M. L. Pace-Savitsky C. (2006). Neuroanatomical correlates of impaired recognition of emotion in dementia. Neuropsychologia 44, 365–373 10.1016/j.neuropsychologia.2005.06.012 16154603

[B96] Rugg M. D. Fletcher P. C. Allan K. Frith C. D. Frackowiak R. S. Dolan R. J. (1998). Neural correlates of memory retrieval during recognition memory and cued recall. Neuroimage 8, 262–273 10.1006/nimg.1998.0363 9758740

[B97] Sarazin M. Pillon B. Giannakopoulos P. Rancurel G. Sansom Y. Dubois B. (1998). Clinicometabolic dissociation of cognitive functions and social behavior in frontal lobe lesions. Neurology 51, 142–148 10.1212/WNL.51.1.142 9674793

[B98] Saxe R. Baron-Cohen S. (2006). The neuroscience of theory of mind. Soc. Neurosci. 1, i–ix 10.1080/17470910601117463 18633783

[B99] Scharmuller W. Ille R. Schienle A. (2013). Cerebellar contribution to anger recognition deficits in Huntington’s disease. Cerebellum 12, 819–825 10.1007/s12311-013-0492-9 23709228 PMC4495283

[B100] Seeley W. W. Crawford R. K. Zhou J. Miller B. L. Greicius M. D. (2009). Neurodegenerative diseases target large-scale human brain networks. Neuron 62, 42–52 10.1016/j.neuron.2009.03.024 19376066 PMC2691647

[B101] Shimazu M. (1985). Revised K-Form Developmental Test. Kyoto: Nakanishi Press.

[B102] Siegal M. Varley R. (2002). Neural systems involved in “theory of mind.” Nat. Rev. Neurosci. 3, 463–471 10.1038/nrn844 12042881

[B103] Singer T. Lamm C. (2009). The social neuroscience of empathy. Ann. N. Y. Acad. Sci. 1156, 81–96 10.1111/j.1749-6632.2009.04418.x 19338504

[B104] Spivak G. (2004). The many faces of Cockayne syndrome. Proc. Natl. Acad. Sci. U.S.A. 101, 15273–15274 10.1073/pnas.0406894101 15494443 PMC524466

[B105] Squire L. R. Ojemann J. G. Miezin F. M. Petersen S. E. Videen T. O. Raichle M. E. (1992). Activation of the hippocampus in normal humans: a functional anatomical study of memory. Proc. Natl. Acad. Sci. U.S.A. 89, 1837–1841 10.1073/pnas.89.5.1837 1542680 PMC48548

[B106] Sridharan D. Levitin D. J. Menon V. (2008). A critical role for the right fronto-insular cortex in switching between central-executive and default-mode networks. Proc. Natl. Acad. Sci. U.S.A. 105, 12569–12574 10.1073/pnas.0800005105 18723676 PMC2527952

[B107] Straube T. Weisbrod A. Schmidt S. Raschdorf C. Preul C. Mentzel H. J. (2010). No impairment of recognition and experience of disgust in a patient with a right-hemispheric lesion of the insula and basal ganglia. Neuropsychologia 48, 1735–1741 10.1016/j.neuropsychologia.2010.02.022 20188112

[B108] Stuss D. Knight R. (2002). Principles of Frontal Lobe Function. New York: Oxford University Press

[B109] Sugita K. Takanashi J. Ishii M. Niimi H. (1992). Comparison of MRI white matter changes with neuropsychologic impairment in Cockayne syndrome. Pediatr. Neurol. 8, 295–298 10.1016/0887-8994(92)90369-A 1388420

[B110] Sugita K. Takanashi J. Suzuki N. Niimi H. (1991). Comparison of cellular sensitivity to UV killing with neuropsychological impairment in Cockayne syndrome patients. Brain Dev. 13, 163–166 10.1016/S0387-7604(12)80023-4 1928608

[B111] Topper R. Mottaghy F. M. Brugmann M. Noth J. Huber W. (1998). Facilitation of picture naming by focal transcranial magnetic stimulation of Wernicke’s area. Exp. Brain Res. 121, 371–378 10.1007/s002210050471 9746143

[B112] Torralva T. Roca M. Gleichgerrcht E. Lopez P. Manes F. (2009). INECO frontal screening (IFS): a brief, sensitive, and specific tool to assess executive functions in dementia. J. Int. Neuropsychol. Soc. 15, 777–786 10.1017/S1355617709990415 19635178

[B113] Vandenberghe R. Price C. Wise R. Josephs O. Frackowiak R. S. (1996). Functional anatomy of a common semantic system for words and pictures. Nature 383, 254–256 10.1038/383254a0 8805700

[B114] Wang K. Hoosain R. Yang R. M. Meng Y. Wang C. Q. (2003). Impairment of recognition of disgust in Chinese with Huntington’s or Wilson’s disease. Neuropsychologia 41, 527–537 10.1016/S0028-3932(02)00171-9 12559147

[B115] Wechsler D. (1997). Wechsler Adult Intelligent Scale III. San Antonio, TX: The Psychological Corp

[B116] Weidenheim K. M. Dickson D. W. Rapin I. (2009). Neuropathology of Cockayne syndrome: evidence for impaired development, premature aging, and neurodegeneration. Mech. Ageing Dev. 130, 619–636 10.1016/j.mad.2009.07.006 19647012

[B117] Weschler D. (1999). Weschler Abbreviated Scale of Intelligence. San Antonio, TX: Psychological Corporation

[B118] Weschler D. (2009). Wechsler Adult Intelligence Scale. New York: Psychological Corporation

[B119] Young A. W. Rowland D. Calder A. J. Etcoff N. L. Seth A. Perrett D. I. (1997). Facial expression megamix: tests of dimensional and category accounts of emotion recognition. Cognition 63, 271–313 10.1016/S0010-0277(97)00003-6 9265872

